# Antiretroviral Drugs Impact Autophagy Differently in Primary Human Astrocytes

**DOI:** 10.3390/cells14231904

**Published:** 2025-12-01

**Authors:** Laura Cheney, Grace McDermott, Hillary Guzik, Joan W. Berman

**Affiliations:** 1 Department of Medicine, Division of Infectious Diseases, Albert Einstein College of Medicine, Bronx, NY 10461, USA; 2 Department of Pathology, Albert Einstein College of Medicine, Bronx, NY 10461, USAjoan.berman@einsteinmed.edu (J.W.B.); 3 Analytical Imaging Facility, Albert Einstein College of Medicine, Bronx, NY 10461, USA; 4 Department of Microbiology and Immunology, Albert Einstein College of Medicine, Bronx, NY 10461, USA

**Keywords:** antiretroviral therapy, astrocytes, BNIP3L/Nix, HIV associated neurocognitive impairment, LC3B, macroautophagy, mitophagy, PINK1-Parkin, p62, selective autophagy

## Abstract

While antiretroviral therapy (ART) has significantly improved the morbidity of HIV infection, ART may contribute to the pathogenesis of HIV associated neurocognitive impairment (HIV-NCI) by interfering with autophagic processes in astrocytes. Autophagy and mitophagy remove unwanted/damaged material and mitochondria from the intracellular environment, respectively. Dysregulated autophagy in astrocytes, abundant CNS cells with crucial homeostatic functions, contributes to many neurodegenerative diseases. Few studies have examined effects of ART on autophagy in astrocytes. We treated primary human astrocytes with a common ART regimen and performed LC3B-II and p62 turnover assays. ART significantly inhibited both LC3B-II and p62 turnover. Since p62, one autophagy receptor that mediates mitophagy, autophagic clearance of mitochondria, turnover was inhibited, we also examined mitophagy. While ART decreased BNIP3L/Nix homodimers, there were no changes in PINK1, Parkin, Mt-CO2, mitochondrial mass, or mitochondria–lysosome colocalization, indicating that ART did not inhibit mitophagy. We show that antiretroviral drugs have distinct effects on autophagic processes in astrocytes, which represents an alteration in their homeostasis, a major function of autophagy. This likely contributes to HIV-NCI. Understanding these impacts is important for improving ART for PWH, who have, by necessity, ongoing ART exposure. It also facilitates development of therapies for HIV-NCI that may include modulation of autophagy.

## 1. Introduction

Autophagy is an evolutionarily conserved, highly regulated, proteolytic process for the degradation of intracellular material in a lysosome-dependent manner. Of the three major forms of autophagy, macroautophagy is the most extensively characterized. In macroautophagy, hereafter called autophagy, intracellular cargo intended for degradation is enclosed within specialized double-membraned vesicles, called autophagosomes (APG). Once enclosed, APG fuse with lysosomes in a process termed maturation, and contents within the APG are degraded. The process from APG formation to maturation is frequently referred to as “flux.” Almost every cell in the human body requires autophagic flux for metabolism and quality control, including long-lived cells such as astrocytes. Astrocytes are abundant central nervous system (CNS) cells with a multifaceted role in CNS homeostasis, and autophagic flux is essential for astrocyte homeostasis [1,2,3,4]. Dysregulated flux contributes to many human diseases [5], and when disrupted in astrocytes, plays a role in Parkinson, Huntington, Alzheimer, Frontotemporal Dementia, and others [4].

Cargo can be incorporated into APG non-specifically, or incorporated in a targeted manner, called selective autophagy. Autophagy receptors, such as Sequestosome-1 (SQSTM1; hereafter called p62), and BCL2/adenovirus E1B 19 kDa protein-interacting protein 3-like (BNIP3L)/Nix, mediate selective autophagy. Receptors either bind to ubiquitin chains that are linked to their cargo, as is the case for p62, or bind directly to their cargo, such as BNIP3L/Nix. Receptors then incorporate their cargo into APG by binding to Microtubule-associated protein 1 light chain 3B-II (LC3B-II), the APG marker. Mitophagy is one form of selective autophagy for the degradation of damaged and dysfunctional mitochondria. A healthy population of mitochondria must be maintained within cells to meet its energy needs, as well as for cell survival. Damaged and dysfunctional mitochondria can induce apoptosis and other cell death pathways [6]. Therefore, mitophagy is important for clearing irreparably damaged mitochondria to avert apoptosis and maintain homeostasis [7,8]. Mitochondrial dysfunction and mitophagy in astrocytes are associated with the pathogenesis of Alzheimer, Parkinson, and Amyotrophic Lateral Sclerosis [9,10,11].

Dysregulation of autophagic processes likely contributes to Human Immunodeficiency Virus (HIV) associated neurocognitive impairment (HIV-NCI). HIV-NCI is a spectrum of motor, behavioral, and cognitive deficits, and develops in 15–50% of people with HIV (PWH) despite having controlled viremia with antiretroviral therapy (ART) [12,13,14,15]. It significantly impacts quality of life, contributes to medication non-adherence, and is an independent risk factor for mortality [16,17,18,19,20,21,22]. The pathogenesis of HIV-NCI in the context of antiretroviral use is not fully characterized. In the pre-ART era, the predominant clinical manifestation of HIV-NCI was dementia [23]. With the advent of ART, the majority of PWH with HIV-NCI experience milder symptoms, ranging from subclinical/asymptomatic impairment to mild-moderate impairment [23,24]. ART inhibits viral replication, reducing the neurotoxic impacts of the virus. That HIV-NCI persists suggests there are other mechanisms that contribute to HIV-NCI that ART cannot quell [24]. Off-target effects of ART itself may contribute to HIV-NCI. While ART has revolutionized the lives of PWH, many antiretroviral drugs are neurotoxic [24,25,26]. Antiretroviral drugs are also known to affect autophagy in many cell types [27,28], but only a few studies have examined the impacts of antiretroviral drugs on autophagy and mitophagy in astrocytes as a possible mechanism of toxicity contributing to HIV-NCI [29,30]. Given the importance of autophagy to astrocyte homeostasis and the link to neurodegenerative diseases when dysregulated, and that ART can affect autophagy, we hypothesized that ART contributes to HIV-NCI by inhibiting autophagic processes in astrocytes.

We studied the impacts of a common ART regimen on autophagy in primary human astrocytes. We show that ART inhibits autophagy at two distinct steps in flux: APG formation as well as APG maturation. We also show that flux of p62, one autophagy receptor that mediates mitophagy, is inhibited. We therefore examined the effects of the same common ART regimen on mitophagy in primary human astrocytes. Our data show that while homodimerization of BNIP3L/Nix, another mitophagy receptor, is decreased, ART does not inhibit mitophagy mediated through this receptor, nor does ART inhibit PINK1-Parkin dependent mitophagy. These findings demonstrate that antiretroviral drugs can impact two autophagic processes differently in primary human astrocytes, and identify a novel mechanism by which antiretroviral drugs may contribute to the pathogenesis of HIV-NCI.

## 2. Materials and Methods

### 2.1. Cells and Antiretroviral Treatments

Primary human cortical astrocytes were isolated as described in [31]. We previously extensively characterized the morphology and function of these cells [32,33,34,35,36,37]. Several different astrocyte donors were used for all experiments. Astrocytes were cultured in phenol-red free DMEM supplemented with 10% FBS and 1% penicillin/streptomycin. Tenofovir (#10199) and emtricitabine (#10071) were obtained from the NIH HIV Reagent Program, Division of AIDS, NIAID, and reconstituted in sterile water. Dolutegravir (#1051375-19-9, Ambeed, Buffalo Grove, IL, USA) was reconstituted in sterile DMSO. Aliquots of antiretroviral stocks were stored at −20 °C and were not subject to freeze–thaw cycles. Cells were treated for 8 h or 24 h, or daily for 7 days, as indicated, with either 5 ng/mL tenofovir + 109 ng/mL emtricitabine + 18 ng/mL dolutegravir (ART), or with a volume of DMSO equivalent to that of dolutegravir as control. The antiretroviral concentrations used are consistent with drug levels achieved in human cerebral spinal fluid [38,39]. Media were replaced fresh before the start of all treatments. For 7 days of daily treatment, media were exchanged on the fourth day of treatment.

### 2.2. Autophagic Flux and Western Blotting

Cells were treated 24 h or daily for 7 days. In the last 2 h or 4 h of treatment, 30 µM Chloroquine (CQ) was added to perform LC3B-II and p62 turnover assays by Western blotting to assess autophagic flux as in Ref. [29] and as recommended in Refs. [40,41,42]. Cell lysates were collected in RIPA buffer containing 1x Halt Protease and Phosphatase Inhibitor Cocktail (Thermo Fisher Scientific, Fair Lawn, NJ, USA). Protein concentration was determined with Protein Assay Reagent Concentrate (Bio-Rad, Hercules, CA, USA) using the Bradford method. Equal amounts of protein, ranging from 30 µg to 70 µg depending on the protein of interest, were subject to SDS-PAGE under reducing conditions, followed by transfer overnight at 4 °C to methanol-activated PVDF. After transfer, total protein optical density (OD) was determined with Revert Total Protein Stain (#926-11021 Li-Cor, Lincoln, NE, USA). Membranes were blocked in 5% non-fat dry milk in TBST, followed by incubation overnight at 4 °C with rabbit anti-LC3B (#2775, Cell Signaling Technology [CST], Danvers, MA, USA), rabbit anti-p62 (#PW9860, Enzo BML, Farimngdale, NY, USA), rabbit anti-PINK1 (#6946, CST), mouse anti-parkin (#ab77924, Abcam, Waltham, MA, USA), rabbit anti-super oxide dismutase 2 (SOD2; #13194, [CST]), rabbit anti-BNIP3L/Nix (#12396, CST), or rabbit anti-mitochondrial encoded cytochrome c oxidase subunit 2 (Mt-CO2; #31219, [CST]) at 1:1000 dilutions in 5% BSA-TBST. HRP-conjugated secondary antibodies were horse anti-mouse (#7076, CST) or goat anti-rabbit (#7074, CST), used at 1:1000 dilution in 5% milk-TBST. Membranes were developed with Super Signal West Femto Chemiluminescent Substrate and Luminol/Enhancer (#34095, Thermo Fisher Scientific) or WesternSure PREMIUM Chemiluminescent Substrate (#926-95010, Li-Cor). When possible, membranes were sequentially reprobed for the various proteins of interest. The Odyssey Fc System (Li-Cor, Lincoln, NE, USA) was used to visualize membranes, and Image Studio v. 5.2 software (Li-Cor) was used for determination of OD. The appearance of individual membranes was optimized in ImageStudio, but optimization does not change the OD of protein bands. For LC3B-II and p62 turnover, i.e., autophagic flux analysis, LC3B-II and p62 were analyzed as in Refs. [29,40,41,42]. Briefly, LC3B-II and p62 OD were normalized to total protein OD of each lane. LC3B-II and p62 steady states are the amount of normalized protein without CQ. APG formation was determined as the difference in normalized LC3B-II in cells treated with CQ for 2 h from LC3B-II with CQ for 4 h. Degradation rate (flux) of LC3B-II and p62 were calculated as the quotient of normalized protein with CQ for 4 h by protein without CQ. Degradation amount (net flux) of LC3B-II and p62 were determined as the difference in protein without CQ from protein with CQ for 4 h. The effect of ART was determined as the fold change in each autophagy parameter relative to DMSO (control). For analysis of all other proteins, the OD of target proteins were normalized to total protein OD of each lane, and the effect of ART was determined as the fold change relative to DMSO (control).

### 2.3. qRT-PCR

Cells were treated for 8 h, 24 h, or daily for 7 days. Total RNA was then isolated with Trizol (#15596026, Thermo Fisher Scientific) according to the manufacturer’s protocol. Equal amounts of RNA were reverse transcribed into cDNA with SuperScript Vilo EZ-DNAse Master Mix (#11766050, Invitrogen, Carlsbad, CA, USA), according to manufacturer’s protocol. Taqman Gene Expression Assays for human *18S* and *p62* (Applied Biosystems, assays Hs99999901_s1 and HS01061917_g1, respectively) were performed using Taqman Gene Expression Master Mix (#4369016, Applied Biosystems) and a QuantStudio 3 Real-Time PCR system (Applied Biosystems, Foster City, CA, USA). Cycling conditions were as recommended for Taqman assays. DEPC-treated water was used as non-template control. The relative quantity of *p62* after ART treatment relative to DMSO (control) was calculated using the 2^−∆∆Ct^ method with *18S* as the reference gene. Efficiencies of *18S* and *p62* PCR reactions were determined with standard curves of untreated astrocyte cDNA to ensure that comparative qRT-PCR analyses of ART-treated and DMSO control cDNA could be performed. Efficiencies of *18S* and *p62* were similar, and within the accepted range of values.

### 2.4. Mitochondrial Mass

Cells were treated for 24 h or daily for 7 days, then trysponized, resuspended in HBSS, and counted in preparation for staining for flow cytometric analyses. Two hundred thousand cells per condition were left unstained, or stained 30 min at room temperature with 100 nM MitoTracker Deep Red (#M22426, Invitrogen) diluted in HBSS, as per manufacturer’s protocol. Cells were fixed in 2% PFA/HBSS. A minimum of 25,000 events per condition were acquired with the Attune NxT flow cytometer (Thermo Fisher Scientific, Fair Lawn, NJ, USA). Forward- and side-scatter voltages were adjusted for optimal acquisition for each astrocyte donor, and the voltage for MitoTracker Deep Red was maintained across all independent experiments. The optimal concentration of Mitotracker Deep Red was determined prior to use. Analyses were performed using FlowJo software v. 10.9.0 (TreeStar, Ashland, OR, USA). The effect of ART was determined as the fold change in mean fluorescence intensity (MFI) relative to DMSO (control). The MFI of unstained cells were negligible.

### 2.5. Mitochondrial Membrane Polarization

Cells were plated in black, clear bottom 96-well plates, and treated for 24 h or daily for 7 days. At the last hour of treatment, 15 µM carbonyl cyanide m-chlorophenyl hydrazone (CCCP; #C2759 [Sigma-Aldrich], St. Louis, MO, USA) was added to some wells as a positive technical control. In the last 30 min of treatment, tetraethylbenzimidazolylcarbocyanine iodide (JC-1; #ab113850 [Abcam]) was added for a final concentration of 10 µM. At treatment end, cells were washed with 1x assay buffer (Abcam), and green and red fluorescence were measured by fluorimetry on a SpectraMax M5 fluorimeter (Molecular Devices, San Jose, CA, USA) with the following excitation/emission wavelengths: green (475/535); red (475/590). Background green and red fluorescence was determined in untreated cells not incubated with JC-1. The ratio of red to green fluorescence minus background fluorescence was calculated, and the effect of ART was determined as a percentage of DMSO (control).

### 2.6. Reactive Oxygen Species

Cells were plated in black, clear bottom 96-well plates, and treated with ART or DMSO for 24 h or daily for 7 days. At treatment end, media were removed and replaced with HBSS containing 10 µM 5(6)-Chloromethyl-2′,7′-dichlorodihydrofluorescein diacetate, acetyl ester (CM-H2 DCFDA; #C6827 [Thermo Fisher Scientific]). Cells were incubated for 1 h, then washed two times with HBSS. ART, DMSO, or 200 µM Pyocyanin (#10009594, Caymen Chemical, Ann Arbor, MI, USA), a positive biological control, diluted in HBSS were then added for an additional hour, after which fluorescence was measured by fluorimetry on a VarioSkan Lux (Thermo Fisher Scientific, Fair Lawn, NJ, USA) with excitation/emission wavelengths of 495/527, respectively. Background fluorescence was determined in untreated cells not incubated with CM-H2 DCFDA. The effects of ART were determined as a percentage of DMSO (control) minus background.

### 2.7. Mitophagic Flux with HaloTag Reporter

A custom-made plasmid encoding HaloTag genetically fused to a mitochondrial targeting sequence (Halo-Mt) was purchased from Promega (proprietary product). The plasmid was packaged into a lentiviral vector for cell transduction in the Genetic Engineering and Gene Therapy Core Facility at Albert Einstein College of Medicine. Cells were transduced, allowed to recover for 24–48 h, then plated onto poly-d-lysine coated glass coverslips (#CLS354086, Corning, Corning, NY, USA). Transduced cells were then treated for 7 days daily with ART or DMSO (control), or 4 h with 10 µM Antimycin A plus 10 µM Oligomycin A (AAO; #75351 [Sigma-Aldrich],). AAO induces mitophagy and is therefore positive controls for the assay. At treatment end, media were removed, cells were washed once with HBSS, and then incubated 30 min in 200 nM Janelia Fluor 549 HaloTag Ligand (#HT1030, Promega, Madison, WI, USA) diluted in HBSS. Cells were then washed once in HBSS, fixed in 2% PFA/HBSS, permeabilized 5 min with 0.01% TritonX-100, then blocked for 30 min at room temperature with block solution described in Ref. [43]. Cells were incubated overnight in a 1:200 dilution of Alexa Fluor 647-conjugated anti-lysosome associated membrane protein 1 (LAMP1, #sc-20011 [Santa Cruz Biotechnology], Dallas, TX, USA) antibody in block solution. Cells were then washed three times with PBS, and mounted onto glass slides with ProLong Diamond Antifade Mountant with DAPI (#P36962, Invitrogen). Three independent experiments were performed. Confocal images were acquired on either a Leica SP8 or a Stellaris 8 confocal microscope (Leica Microsystems, Wetzlar, Germany), using the 63x oil objective 1.4na. 20–24 images of individual cells for each condition (ART or DMSO) per each independent experiment were taken. Microscope settings were established for each independent experiment, and each condition within each experiment was imaged identically and obtained using the same microscope. Images were analyzed using Volocity Quantitation software v 6.5.1 (Quorum Technologies, Lewes, UK). Contrast adjustments for individual channels were applied identically across all images within the independent experiments. Briefly, the mean number of red (mitochondria) and green (pseudocolor; lysosomes) objects were quantified in the cytosol using the “measure object” tool after establishing appropriate fluorescent thresholds for object detection. Using the “exclude non-touching” tool, the volume (µm^3^) of red objects colocalized to green objects was determined, then normalized to the total volume of green objects in the cytosol per cell. The effects of ART were determined as the mean fold change in normalized red/green colocalization relative to DMSO (control). All images were captured and analyzed in a blinded fashion.

### 2.8. Mitochondrial Network Analysis (MiNA)

To assess mitochondria network morphology, MiNA was performed with the ImageJ v. 2.17 macro toolset, MiNA v. 3.0 [44], on the confocal images obtained for colocalization. The effects of ART were determined as the mean fold change in the mitochondria area in µm^2^ per cell area in µm^2^ (Footprint), the mean number of branches per individual mitochondrial network, and the mean branch length in µm of all lines representing mitochondrial structures. MiNA was performed in a blinded fashion.

### 2.9. Statistical Analyses

Data were analyzed using GraphPad Prism software v. 10.1.2 (GraphPad Software, LLC, Boston, MA, USA). Normality of data was tested using the D’Agostino and Pearson normality test. For fold-changes, comparisons were made between ART and DMSO (control) which was set to a theoretical mean of 1, by one-Sample *t*-tests for normally distributed data, or by Wilcoxon Signed Rank tests for data not normally distributed. For percents of control, the same comparisons were made with DMSO (control) set to 100, and statistical tests were performed according to the normality of the data. *p* values of <0.05 were considered significant.

## 3. Results

### 3.1. ART Inhibits Autophagy

**Figure 1:**
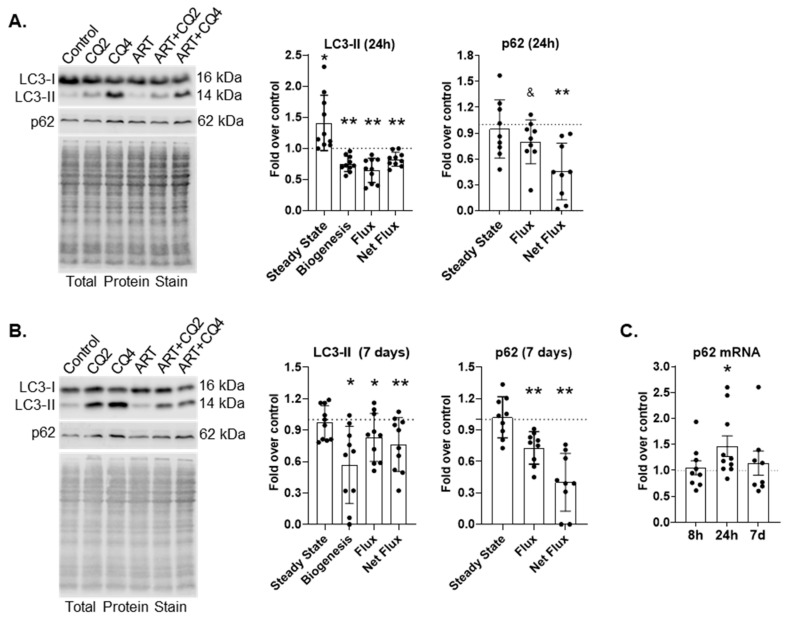
ART inhibits macroautophagy in astrocytes. (**A**,**B**) Primary human astrocytes were treated for 24 h or daily for 7 days with ART, and LC3-II and p62 turnover was assessed by Western blotting. Representative LC3 and p62 Western blots after (**A**) 24 h or (**B**) 7 days of daily ART. Left graphs show the mean fold changes in LC3-II steady state, biogenesis, flux and net flux after (**A**) 24 h and (**B**) 7 days of daily ART. Right graphs show the mean fold changes in p62 steady state, flux and net flux after (**A**) 24 h and (**B**) 7 days of daily ART. Control is represented by the dashed lines at 1. Error bars depict SD. n = 9–10; * *p* < 0.05 by One Sample *t* test; ** *p* < 0.005 by One Sample test; and & *p* < 0.05 Wilcoxon signed rank test. (**C**) Primary human astrocytes were treated for 8 h, 24 h, or daily for 7 days with ART, then qRT-PCR for *p62* was performed. Graph shows mean fold changes relative to control represented by the dashed line at 1. Error bars signify SEM. n = 8–10; * *p* < 0.05 by One Sample *t* test.

ART may contribute to HIV-NCI by inhibiting autophagy in astrocytes, cells important for CNS homeostasis. We previously showed that a combination of tenofovir, emtricitabine, and raltegravir inhibits autophagy in primary human astrocytes [29]. To determine whether a more contemporary antiretroviral regimen of tenofovir, emtricitabine and dolutegravir also inhibits autophagy, we performed LC3B-II turnover assays as described in Methods and elsewhere [29,40,41,42]. We treated astrocytes for either 24 h or 7 days daily with ART, then used chloroquine (CQ) for either the last 2 h or 4 h to inhibit lysosome degradation. We measured LC3B-II by Western blotting to determine APG formation, and also rate (flux) and amount (net flux) of APG maturation. Treatment of 24 h with ART significantly increased the steady state of LC3B-II by 140% relative to control (*p* < 0.05; Figure 1A). This increase was due to significantly decreased rate (flux) and amount (net flux) of maturation. Flux was decreased 35% (*p* < 0.005; Figure 1A), and net flux was decreased 20% (*p* < 0.005; Figure 1A) relative to control. Also, 24 h ART significantly decreased APG formation by 25% relative to control (*p* < 0.005; Figure 1A). These data suggest that ART causes two defects in autophagic flux: APG formation as well as APG maturation.

After 7 days of daily ART, there was no change in LC3B-II steady state relative to control (Figure 1B). However, formation, flux, and net flux were significantly decreased. Formation was decreased by 50% (*p* < 0.05), flux was decreased by almost 20%, and net flux was reduced by 25% (*p* < 0.05 and *p* < 0.005, respectively) relative to control (Figure 1B). These data indicate that both defects caused by ART after 24 h persist after 7 days of daily ART.

p62 is an autophagy receptor that transports polyubiquitinated cargo to forming APG. p62 is incorporated into the inner membrane of the APG and is degraded after APG maturation similar to the LC3B-II that is present on the inner APG membrane. It can be used in conjunction with LC3B-II to confirm changes in autophagy dynamics. We performed p62 turnover assays by Western blotting after 24 h or 7 days of daily ART. p62 flux and net flux were significantly decreased after 24 h ART (20%, *p* < 0.05 and 55%, *p* < 0.005, respectively;Figure 1A). p62 flux and net flux were also decreased after 7 days of daily ART (Figure 1B). Flux was decreased by almost 30% (*p* < 0.005), and net flux was decreased by 60% (*p* < 0.005) relative to control. To confirm that decreased p62 turnover was due to inhibited autophagic flux and not due to decreased p62 transcription, we performed qRT-PCR for *p62* after 8 h, 24 h, or 7 days of daily treatment. There was no difference in *p62* message relative to control after 8 h or 7 days of daily ART (Figure 1C). There was a significant 1.5-fold increase in *p62* message after 24 h ART (*p* < 0.05; Figure 1C). These confirm p62 turnover is decreased because of inhibited autophagic flux and not due to decreased transcription. Decreased p62 turnover supports our LC3-II turnover findings, confirming that autophagy is inhibited by ART.

To ensure that autophagy changes caused by ART were not related to cytotoxicity of ART, we performed LDH assays by fluorimetry after 24 h and 7 days of daily ART. There was no significant LDH release from cells treated with ART at either treatment time point (Figure S1).

### 3.2. ART Does Not Inhibit PINK1-Parkin Mediated Mitophagy

Selective autophagy is the process by which specific intracellular components are targeted for autophagic degradation. Mitophagy, one form of selective autophagy, is the selective recognition and autophagic degradation of mitochondria. Mitophagy is regulated in either a ubiquitin-dependent or receptor-mediated manner. In the former, mitochondrial degradation is promoted by the ubiquitination of several outer mitochondrial membrane proteins which are then recognized by autophagy receptors, such as p62, that bind ubiquitin and facilitate incorporation into forming APG. In the latter, mitophagy is mediated by protein receptors that reside on the mitochondrial membrane that bind directly to LC3B-II, such as BNIP3L/Nix. Since p62 participates in mitophagy, and ART decreases p62 flux in primary human astrocytes (Figure 1A,B), we hypothesized that ART also inhibits mitophagy in primary human astrocytes.

**Figure 2:**
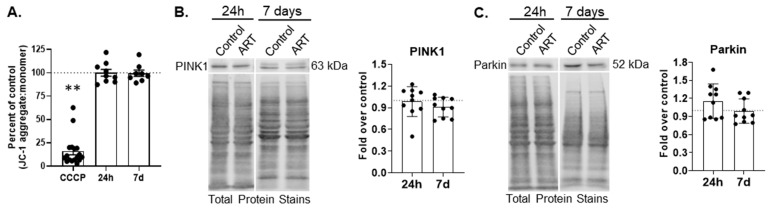
ART does not change mitochondrial membrane polarization, nor PINK1 or Parkin levels in astrocytes. (**A**) Primary human astrocytes were treated for 24 h or daily for 7 days with ART, or with 15 µM CCCP for 1 h, then dyed with JC-1. Fluorescence was measured by fluorimetry. Graph shows the mean percent changes in JC-1 aggregate to monomer ratio, which signifies the degree of mitochondrial membrane polarization, after 24 h or 7 days of daily ART, relative to control represented by the dashed line at 100. Error bars represent SEM. n = 8–9 for ART, and n = 17 for CCCP as CCCP values for all experiments were combined for graphical representation. ** *p* < 0.0001 by Wilcoxon Signed Rank test. (**B**,**C**) Primary human astrocytes were treated for 24 h or daily for 7 days with ART, then PINK1 and Parkin Western blotting was performed. Representative Western blots after 24 h and 7 days of daily ART are shown. Graphs are the mean fold changes in (**B**) normalized PINK1 and (**C**) Parkin after 24 h or 7 days of daily ART, relative to control shown by the dashed line at 1. Error bars represent SD. n = 10.

Ubiquitin-dependent mitophagy is mediated by PTEN-induced putative kinase 1 (PINK1)-Parkin. In PINK1-Parkin mitophagy, mitochondrial membrane depolarization stabilizes PINK1 on the outer mitochondrial membrane (OMM), leading to the recruitment and activation of Parkin, an E3 ubiquitin ligase that builds ubiquitin chains on proteins located on the OMM. This ultimately recruits autophagy receptors that recognize polyubiquitin chains, such as p62, that then incorporate mitochondria into APG. To determine whether ART causes mitochondrial membrane depolarization, we treated cells for 24 h or daily for 7 days, and measured polarization by fluorimetry. CCCP was used as a positive control. One-hour CCCP treatment significantly decreased mitochondrial membrane polarization, as expected (*p* < 0.001; Figure 2A). ART did not change membrane polarization relative to control after either 24 h or 7 days of daily treatment (Figure 2A). While mitochondrial membrane polarization was not affected by ART, ART could still impact PINK1-Parkin mediated mitophagy. Therefore, we measured PINK1 and Parkin by Western blotting after 24 h and 7 days of daily ART. There was no change in the relative amount of PINK1 or Parkin at either ART time point (Figure 2B,C). These data suggest that ART does not depolarize mitochondrial membranes, and that ART does not induce nor inhibit PINK1-Parkin mediated mitophagy.

### 3.3. ART Inhibits BNIP3L/Nix Homodimerization

**Figure 3:**
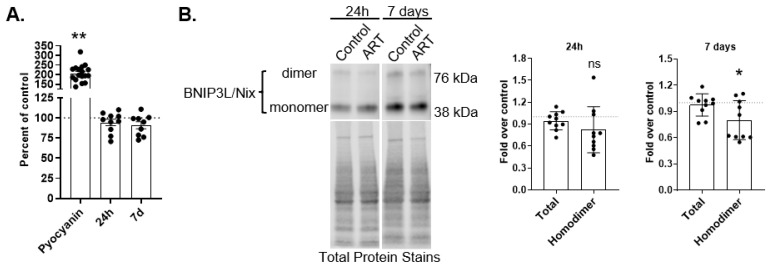
ART does not generate ROS, but decreases BNIP3L/Nix homodimer in astrocytes. (**A**) Primary human astrocytes were treated for 24 h or daily for 7 days with ART, or with 200 μM Pyocyanin for 1 h, then dyed with CM-H2 DCFDA. Fluorescence was measured by fluorimetry. Graph shows the mean percent ROS after 24 h or 7 days of daily ART, relative to control represented by the dashed line at 100. Error bars represent SEM. n = 9–10 for ART, and n = 17 for Pyocyanin as Pyocyanin values for all experiments were combined for graphical representation. ** *p* < 0.0001 by One Sample *t* test. (**B**) Primary human astrocytes were treated for 24 h or daily for 7 days with ART, then BNIP3L/Nix Western blotting was performed. Representative BNIP3L/Nix Western blots after 24 h and 7 days of daily ART treatment are shown. Graphs are the mean fold changes in normalized BNIP3L/Nix after 24 h ART (left) and after 7 days of daily ART (right), relative to control shown by the dashed line at 1. Error bars represent SD. n = 10. ns = not significant; * *p* < 0.05 by One Sample *t* test.

BNIP3L/Nix is one receptor for receptor-mediated mitophagy. BNIP3L/Nix is an OMM protein that, when senses distinct cellular and mitochondrial signals, will homodimerize to recruit APG by binding directly to LC3B-II [45]. While the molecular mechanisms underlying BNIP3L/Nix mediated mitophagy are not fully described, increases in cytosolic or mitochondrial reactive oxygen species (ROS) can induce mitophagy through this receptor [46]. To determine whether ART increases ROS, we measured general ROS by fluorimetry after treatment with ART for 24 h or daily for 7 days. There was no difference in ROS production at either ART time point compared to control (Figure 3A). We also analyzed the amount of superoxide dismutase 2 (SOD2) by Western blotting after 24 h and 7 days of daily ART. SOD2 converts superoxide into hydrogen peroxide within the mitochondrial matrix, thereby protecting against oxidative damage within the mitochondria. We found no difference in SOD2 relative to control at either time point (Figure S2). These data indicate that ART does not induce cellular ROS, nor does ART change SOD2 amount, suggesting that ROS could be adequately neutralized within the mitochondria.

While we did not detect changes in general ROS after ART, ART could still impact BNIP3L/Nix mediated mitophagy. We examined total BNIP3L/Nix by Western blotting after 24 h and 7 days of daily ART. There was no difference in total BNIP3L/Nix relative to control after either ART treatment period (Figure 3B). BNIP3L/Nix homodimerization is important for mitophagy to proceed [45]. We also examined BNIP3L/Nix homodimer by Western blotting after both ART treatment time periods. There was an 18% decrease in BNIP3L/Nix homodimer 24 h (trend; Figure 3B), and a significant 20% decrease in BNIP3L/Nix homodimer after 7 days of daily ART (*p* < 0.05; Figure 3B). The decreased BNIP3L/Nix homodimer suggests that ART may inhibit mitophagy mediated by this receptor without inducing any stimuli for mitophagy such as ROS.

### 3.4. ART Does Not Inhibit Mitophagy

**Figure 4:**
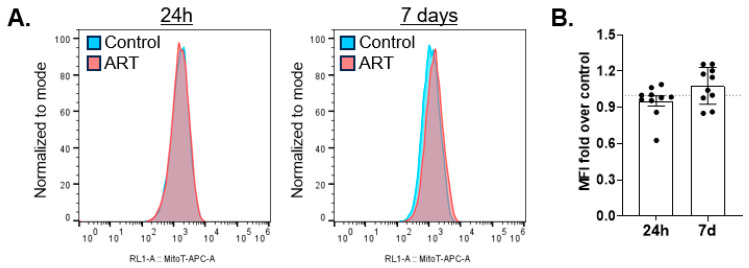
ART does not increase mitochondrial mass. Primary human astrocytes were treated for 24 h or daily for 7 days with ART, stained with Mitotracker Deep Red, and acquired by flow cytometry. (**A**) Representative histograms of 24 h (left) and 7 days of daily ART (right). (**B**) Mean fold change in mean fluorescence intensity (MFI) after 24 h or 7 days of daily ART relative to control shown by the dashed line at 1. Error bars depict SEM. n = 10.

BNIP3L/Nix homodimer is decreased by ART, suggesting that ART may inhibit mitophagy mediated by this receptor. If so, this could be evidenced by increased mitochondrial mass. We treated astrocytes with ART for 24 h or daily for 7 days, and assessed mitochondrial mass by flow cytometry with Mitotracker Deep Red. There was no change in mitochondrial mass relative to control after either 24 h or 7 days of daily ART (Figure 4A,B).

We also assessed the mitochondrial network, the interconnected, branched mitochondria system within a cell, after ART treatment using the macro toolset Mitochondrial Network Analysis (MiNA) for ImageJ [44]. There was no difference in the mitochondrial footprint, supporting the flow cytometry data (Figure S3A). There was also no difference in the mean number of network branches or in length of branches per network (Figure S3B and C, respectively). Antimycin A plus Oligomycin A (AAO) treated cells, a positive control, were analyzed similarly. AAO decreased all three parameters, as expected (Figure S5C–E).

**Figure 5:**
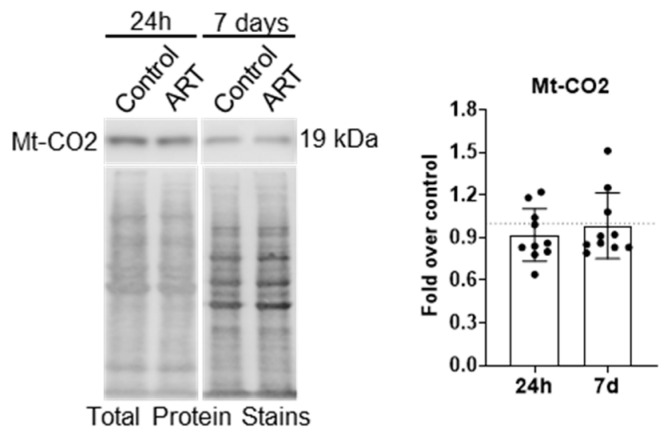
ART does not increase Mt-CO2 levels in astrocytes. Primary human astrocytes were treated for 24 h or daily for 7 days with ART, then Mt-CO2 Western blotting was performed. Representative Mt-CO2 Western blots are shown. Graph shows the mean fold changes in normalized Mt-CO2 relative to control shown by the dashed line at 1. Error bars depict SD. n = 10.

Unlike many OMM proteins, which are degraded by the proteosome in addition to mitophagy, mitochondrial matrix proteins are degraded by mitophagy only. An increase in matrix proteins may be evident if ART inhibits mitophagy. Therefore, we performed Western blotting for mitochondrially encoded cytochrome c oxidase subunit 2 (Mt-CO2), a component of the electron transport chain that resides in the mitochondrial matrix, after treating astrocytes for 24 h or daily for 7 days with ART. There was no change in Mt-CO2 relative to control at either treatment time point (Figure 5).

**Figure 6:**
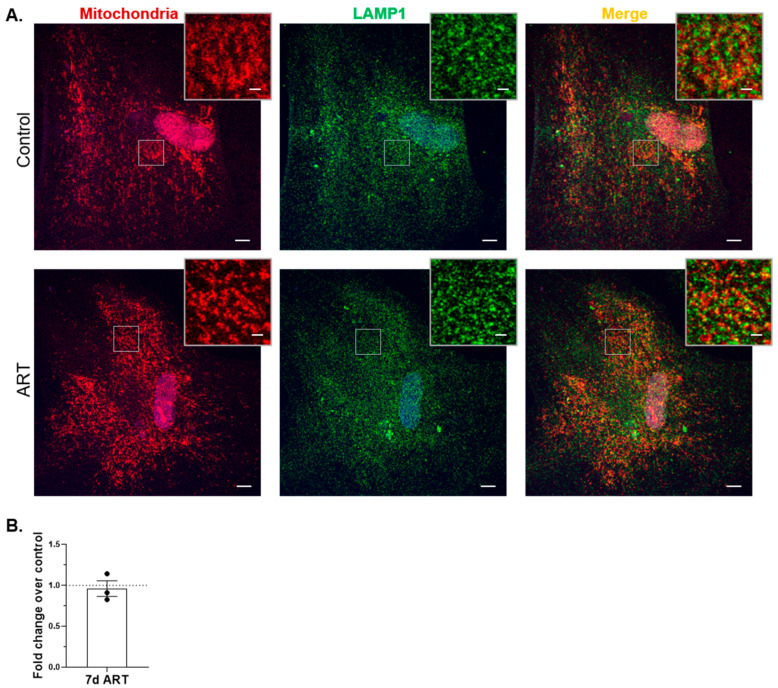
ART does not inhibit mitophagy in astrocytes. Primary human astrocytes expressing Halo-Mt were treated daily for 7 days with ART, exposed to HaloTag ligand, fixed, and then immunolabeled with LAMP1. Cells were imaged by confocal microscopy, and analyzed for mitochondrial colocalization with LAMP1 as described in Methods. (**A**) Representative images of control and ART-treated cells. Red = mitochondria; green (pseudocolor) = lysosomes; blue = nucleus. Scale bar = 10 µM. Insets are enlarged views of the boxed areas, highlighting mitochondrial morphology (left panels), and colocalization (right panels); inset scale bar = 2 µM. (**B**) Graph is the mean fold change of mitochondria colocalized to lysosomes normalized to total lysosomes, relative to control shown by the dashed line at 1. Error bars represent SEM. n = 3 independent experiments, with a total of 60–68 cells per condition analyzed.

Flow cytometry for mitochondrial mass and Mt-CO2 Western blotting may not be sufficiently sensitive to detect low-level mitophagy inhibition caused by ART. To examine mitophagic flux more sensitively in ART-treated astrocytes, we used a HaloTag-based mitophagy reporter to colocalize mitochondria with lysosomes after ART treatment. Briefly, astrocytes were transduced to express HaloTag fused to a mitochondrial targeting sequence. The astrocytes expressed the HaloTag construct, and it localized properly to the mitochondrial membrane (Figure S4). Cells were treated with ART daily for 7 days, or 4 h with 10 µM Antimycin A plus 10 µM Oligomycin A (AAO), then exposed to HaloTag ligand, fixed, and immunolabeled with anti-lysosome associated membrane protein 1 (LAMP1), a lysosome marker. Colocalization of mitochondria (red) with LAMP1 (pseudocolor green) was assessed by confocal microscopy. Decreased mitophagic flux would be represented by decreased yellow (merge) fluorescence. AAO induced mitophagic flux, demonstrating an increase in colocalization of mitochondria with lysosomes (Figure S5A,B). There was no significant difference in mitochondrial colocalization with lysosomes after 7 days of daily ART, relative to control (Figure 6A,B), indicating ART does not inhibit mitophagy.

## 4. Discussion

Autophagy is vital for cell homeostasis, providing building blocks for energy, and eliminating damaged organelles, protein aggregates, and other noxious materials. Autophagy also participates in the regulation of many cell processes including the turnover of the proteome during neurogenesis, glutamate uptake, and redox balance, all of which can affect cell survival [47,48,49,50]. When dysregulated, autophagy contributes to the pathogenesis of many neurodegenerative diseases and likely contributes to HIV-NCI [29,51,52,53,54,55,56,57]. While antiretroviral drugs have significantly improved the clinical manifestations of HIV-NCI, the mechanisms that underly the pathogenesis of HIV-NCI in the current ART era remain incompletely characterized. We show for the first time that a common antiretroviral regimen of tenofovir, emtricitabine, and dolutegravir, used in physiologically relevant concentrations [38,39], inhibits autophagy, but does not inhibit mitophagy, in primary human astrocytes. We identified an understudied off-target effect of commonly prescribed antiretroviral drugs and describe their differential impacts on autophagic processes in human astrocytes. Autophagy dysregulation represents a perturbation in homeostasis, a major function of autophagy. We propose that these effects on autophagy, especially after long-term exposure, contribute to HIV-NCI in the current era of antiretroviral therapy.

We found that the combination of tenofovir, emtricitabine, and dolutegravir decreased APG formation as well as degradation, with short-term (24 h) and longer-term (7 days daily) exposure models. The mechanism(s) by which ART inhibits both APG formation and maturation are not well defined. It is possible that ART inhibits APG formation independently of APG maturation by acting on factors that coordinate only one step. ART may inhibit APG formation by decreasing ATG5, which is necessary for catalyzing the lipidation of LC3B-I to create LC3B-II. Preliminary data from our laboratory suggests that the ART regimen used in these studies decreases ATG5 protein. ART could inhibit APG maturation independently of formation by interfering with proteins that mediate fusion with lysosomes, such as vesicle-associated membrane protein 8 (VAMP8), the tethering protein synaptosome-associated protein 29 (SNAP29), or the tethering homotypic fusion and vacuole protein sorting (HOPS) complex. To our knowledge, there are no studies examining the effects of antiretroviral drugs on these proteins. Alternatively, ART could interfere with lysosome function, thereby mimicking a maturation defect. Bictegravir, an integrase inhibitor like Dolutegravir, was shown to deacidify lysosomes and decrease lysosomal degradation in primary rat oligodendrocyte precursor cells [58]. Another study of primary rat microglia demonstrated that a combination of tenofovir disoproxil fumarate, emtricitabine, and dolutegravir induced lysosome membrane permeabilization with increased lysosomal pH, and decreased cathepsin D activity [59], a lysosome aspartic protease linked to several neurodegenerative diseases [60]. Preliminary data from our laboratory indicate that Cathepsin D levels in astrocytes are unchanged by ART. It is also possible that ART impacts a single factor that participates in both APG formation and maturation. *BECN1*/Beclin 1, a scaffold protein in the PI3K complex required for APG formation, also forms a separate PI3K complex with vacuolar protein sorting 34 (VSP34) and UV radiation resistance associated gene (UVRAG) that facilitates APG maturation [61]. One study showed that *BECN1* transcripts were decreased in peripheral blood mononuclear cells from PWH who were virally suppressed with various antiretroviral regimens for a median of 8.4 years [62], suggesting antiretroviral drugs can change *BECN1* transcription. Preliminary data from our lab suggests that the ART used in our studies decreases Beclin-1. The soluble NSF attachment protein receptor (SNARE) Syntaxin 17 (STX17) also participates in both APG formation and maturation. STX17, along with SNAP29 and VAMP8, coordinates APG fusion with lysosomes, and also binds ATG14 at the ER membrane to facilitate biogenesis of the APG membrane [63,64]. There are no studies that have examined the possible effects of antiretroviral drugs on STX17.

We also do not know whether autophagic flux is inhibited by a single antiretroviral agent, if individual agents act independently on steps in autophagy, or if it is the combination of the three drugs mediating inhibited flux. It is important to characterize the effects of combinations of antiretroviral drugs to understand the biological experience of PWH on ART since current treatment regimens comprise combinations of two or three drugs. It is also important to know the effects of individual antiretroviral drugs on autophagy [27,28]. This is underscored by the difference in effects on autophagy reported in this study compared to the effects of the combination of tenofovir, emtricitabine, and raltegravir as detailed in our prior study [29]. The cocktail with raltegravir inhibited APG formation only, whereas both formation and degradation were inhibited by the cocktail containing dolutegravir used in the current study. Raltegravir and dolutegravir are integrase strand transfer inhibitors (ISTI). These suggest that ISTI are mediating the autophagy inhibition, and that different ISTI have different impacts on autophagy. ISTI have been associated with HIV-NCI [65,66,67]. Determining the specific proteins involved in the autophagy defects resulting from either combinations of or individual drugs would increase our understanding of ART’s effects on HIV-NCI pathogenesis and could facilitate the development of new antiretroviral drugs with fewer off-target affects, or modulators of autophagy that could benefit PWH with HIV-NCI. The mechanism(s) by which ART inhibits autophagic flux in astrocytes are the focus of current studies.

We also found that ART inhibited p62-mediated selective autophagy, and ART also decreased BNIP3L/Nix homodimerization. However, these effects did not confer an inhibition in mitophagy. Mitochondrial membrane depolarization can lead to ubiquitin-dependent mitophagy through PINK1-Parkin and p62. In our studies, ART neither depolarized mitochondrial membranes, nor changed levels of PINK1 or Parkin, suggesting ART did not impact ubiquitin-dependent mitophagy mediated by these proteins. We did not examine the impacts of ART on other autophagy receptors that are similar to p62, such as neighbor of BRCA1 gene 1 (NBR1), nuclear dot protein 52 kDa/calcium-binding and coiled-coil domain-containing protein 2 (NDP52/CALCOCO2), tax-1 binding protein 1 (TAX1BP1) or optineurin (OPTN). It is possible that these receptors compensate for each other when one is inhibited, although the typical signal for ubiquitin-dependent mitophagy, mitochondrial membrane depolarization, was not induced by ART, nor were PINK1 or Parkin affected.

Other intracellular insults, such as excess ROS, can induce BNIP3L/Nix mediated mitophagy. We found that ART does not generate excess ROS in astrocytes, and ART decreased homodimers of BNIP3L/Nix after 24 h and significantly so after 7 days of treatment. While the decreased BNIP3L/Nix homodimers suggest ART may inhibit BNIP3L/Nix mediated mitophagy, ART did not increase mitochondrial mass, nor levels of Mt-CO2 at either time point of treatment, nor decrease the colocalization of mitochondria with lysosomes after 7 days of treatment. These indicate that although BNIP3L/Nix homodimerization was decreased, mitophagy was not inhibited by ART. We did not determine the impacts of ART on other mitophagy receptors such as FUN14 domain containing 1 (FUNDC1) [68], which may compensate for BNIP3L/Nix. It was shown that knockdown of BNIP3L/Nix in differentiating cardiac progenitor cells increased FUNDC1 transcripts, and vice versa, in an adult mouse model of ischemia [69]. Therefore, it is possible that ART inhibits BNIP3L/Nix mediated mitophagy, but we could not detect this due to compensation by FUNDC1. While there is partial overlap in the stimuli that activate mitophagy receptors, and some studies suggest there is some functional redundancy amongst mitophagy receptors, data is currently limited, and compensation is likely dependent on physiological context and receptor expression patterns in specific cell types [70,71].

It is also possible that mitophagy is inhibited more evidently after longer periods of ART exposure. This is potentially illustrated by our autophagic flux data. While the 24 h and 7 days of daily ART treatments cannot be directly compared because of the experimental design, that LC3B-II steady state is increased after 24 h but unchanged from control after 7 days of daily ART suggests that the defect in APG formation is increasingly more profound as duration of ART exposure increases. Current antiretroviral drugs control HIV replication; they cannot cure HIV, necessitating lifelong treatment. Therefore, PWH experience long-term antiretroviral exposure. The decrease in BNIP3L/Nix homodimers may intensify with longer ART treatment, leading to frank mitophagic flux inhibition.

Mitophagy receptors play a role in mitochondrial fission and fusion. Fission and fusion regulate the mitochondrial network to meet the metabolic demands of the cell and maintain homeostasis. It was shown that knockdown of BNIP3L/Nix plus FUNDC1 in adult mouse cardiac progenitor cells resulted in fragmented, punctate, and dysfunctional mitochondria [69]. It is not known whether BNIP3L/Nix homodimerization is important for mitochondrial fission. If so, the decrease in BNIP3L/Nix homodimers caused by ART may impact fission in astrocytes, which could ultimately result in abnormal mitochondrial morphology and dysfunction. However, we did not detect gross changes in the mitochondrial network with MiNA.

While ART did not inhibit mitophagy, it is possible that ART inhibits other forms of selective autophagy mediated through p62. For example, ART may inhibit lipophagy, the degradation of lipids. Dysregulated lipophagy contributes to lipodystrophy, abnormal fat distribution in the body, which was a common side effect of early antiretroviral drugs. Lipodystrophy occurs much less frequently with modern antiretroviral drugs; however, lipophagy could still be inhibited during inhibited p62 flux. This would impact lipid metabolism in astrocytes and could contribute further to HIV-NCI. Alternatively, certain forms of selective autophagy may be prioritized over others during ART exposure. Recent studies indicate that the degradative capacity of selective autophagy as a whole is limited. In a model of Zellweger Spectrum Disorders, a group of rare, inherited diseases characterized by peroxisome loss, increased pexophagy, the degradation of peroxisomes, resulted in decreased aggrephagy, the degradation of protein aggregates, and mitophagy [72]. The pathological upregulation in pexophagy with resulting decreased aggrephagy and mitophagy caused protein aggregates and damaged mitochondria to accumulate [72]. When mitophagy was experimentally increased, pexophagy decreased [72]. These highlight that with an inherent limited capacity, a lack of balance amongst the forms of selective autophagy can contribute to pathogenic processes. Current studies in our lab are focused on the impacts of ART on other forms of selective autophagy in astrocytes.

There is excess ROS in HIV infection, impaired antioxidant responses in PWH, and oxidative stress plays a role in HIV-NCI neuropathogenesis [73,74,75,76,77,78]. p62 links autophagy to the antioxidant response by interacting with the E3 ubiquitin ligase adaptor Kelch-like ECH-associated protein 1 (Keap1). Keap1 facilitates the constitutive proteasomal degradation of nuclear factor erythroid 2-related factor 2 (NRF2), a transcription factor that regulates gene expression for antioxidant defense. p62, especially when accumulated, can compete with NRF2 for its binding site on Keap1 with subsequent translocation of NRF2 into the nucleus to coordinate cytoprotective gene expression [79]. NRF2 and p62 exist in a positive feedback-loop as p62 is a gene target of NRF2 [80]. The significant increase in p62 mRNA after 24 h ART suggests that the NRF2 antioxidant response was activated, despite there being no accumulation of p62 protein. This may have caused the lack of excess ROS detection, indicating that at least in the short-term, astrocytes can mitigate ROS. Nonetheless, ART inhibited p62 flux. It is possible that this inhibition, after long term ART exposure, may dysregulate the NRF2-Keap1 antioxidant system such that the activation of important cytoprotective genes is impaired when needed.

One limitation of our study is that we did not determine whether ART induces mitochondrial ROS (mtROS). After extensive troubleshooting of multiple assays, we were unable to reliably detect mtROS. That SOD2 was unchanged by ART suggests that astrocyte mitochondria would neutralize mtROS adequately, should it be generated, although we did not examine SOD2 function. Another limitation is that we did not examine the effects of this antiretroviral regimen in the context of HIV infection. We previously showed that APG maturation was increased in astrocytes treated with the HIV protein Nef. APG formation was decreased after treatment with a combination of tenofovir, emtricitabine and raltegravir. Concomitant treatment with Nef plus the antiretroviral cocktail caused both defects: APG biogenesis was inhibited and APG maturation was increased [29]. This underscores the importance of examining the effects of antiretroviral drugs independently from whole virus or viral proteins because they impart their own impacts on autophagic processes in astrocytes. It is controversial whether astrocytes are productively infected with HIV [81]. Regardless, astrocytes are exposed to viral proteins [82,83,84], HIV itself effects autophagy in CNS cells, and autophagy changes are associated with HIV neuropathogenesis [51,52,53,54,55,56,57]. It is possible that mitophagy in astrocytes is impacted by viral proteins such as Nef, which is produced even in the presence of ART [85,86,87]. Antiretroviral drugs do not directly target Nef. Therefore, it would be expected that ART would not mitigate any effect on mitophagy caused by Nef. Additionally, impacts on autophagic processes caused by viral proteins may be compounded by ART, as shown previously and in this discussion. It is important to continue studies that determine the effects of antiretroviral drugs plus viral proteins/whole virus on autophagy in astrocytes to broaden the understanding of the pathogenesis of and develop treatment for HIV-NCI for PWH.

Astrocytes are key homeostatic cells in the CNS that rely on autophagy for homeostasis. Our studies indicate that ART has distinct effects on autophagic processes in astrocytes, representing an important change in astrocyte physiology, that after prolonged exposure, may contribute to the pathogenesis of HIV-NCI. Autophagy also contributes to the regulation of other supportive functions that astrocytes perform for the CNS macro- and microenvironments. ART-induced autophagy inhibition may also reduce astrocyte capacity to perform other functions, thereby contributing further to HIV-NCI. These are also the focus of ongoing studies in our laboratory. In addition, major pre-clinical and clinical efforts have been devoted to autophagy drug discovery, with small successes being demonstrated for some neurodegenerative conditions [88]. Studies of ART in the context of HIV-NCI will identify possible avenues for therapeutic development for HIV-NCI, possibly through modulation of autophagy.

It has also been hypothesized that autophagy could be manipulated in concert with cell death pathways to assist in the elimination of viral reservoirs [89]. Viral reservoirs persist in many tissues in the body, including in the CNS, despite ART. Persistence is a major barrier to HIV cure. ART-induced inhibition of autophagy in astrocytes could potentially thwart a therapeutic strategy for cure that targets autophagy. Expanding the knowledge of the effects of antiretroviral drugs on autophagy in astrocytes will expand our knowledge of the pathogenesis of HIV-NCI in the current ART era and potentially direct the development of broader therapeutic strategies for treating and curing HIV.

## Data Availability

The raw data supporting the conclusions of this article will be made available by the authors upon reasonable request.

## Supplementary Materials

The following supporting information can be downloaded at: https://www.mdpi.com/article/10.3390/cells14231904/s1. Figure S1: ART is not cytotoxic to astrocytes. Figure S2: ART does not change SOD2 in astrocytes. Figure S3: ART does not alter the mitochondrial network. Figure S4: Halo-Mt localizes properly to the mitochondrial membrane. Figure S5: Mitophagic flux can be detected using Halo-Mt, and Antimycin A plus Oligomycin O reduce the size of the mitochondrial network.
