# Supplementary material for: Antiretroviral Drugs Impact Autophagy Differently in Primary Human Astrocytes
Source: Cells. 2025 Dec 1;14(23):1904. doi: 10.3390/cells14231904 (PMC12691388; doi:10.3390/cells14231904)
Supplement: Supplementary file 1 [file cells-14-01904-s001.zip › cells-3963921-SI.pdf]

Supplemental Methods

Cytotoxicity: CytoTox-ONE Homogeneous Membrane Integrity Assays (Promega, #G7890) were performed on cell supernatants. Cells were plated into black, clear bottom 96-well plates in phenol red free Dulbeco's Modified Eagle Medium, and treated with ART or DMSO for 24 h or 7 days daily. Supernatants were collected at treatment end, and added into a separate black, clear bottom 96-well plate. For 7-day daily treated cells, supernatants were collected prior to the media exchange described for 7-day daily treatments, and stored at -20°C until the end of the 7-day treatment period, at which point it was combined with supernatant collected at treatment end. Supernatants from cells lysed immediately before treatment end with 9% Triton-X 100 were collected to determine maximum LDH release. The assay was performed as per the manufacturer's protocol by fluorimetry on the VarioSkan Lux with excitation/emission wavelength of 560/590. Lactate dehydrogenase (LDH [Millipore], #L1254) was included as a positive technical control (data not shown). Media alone was also included to determine baseline LDH amount. The effects of ART and DMSO were determined as a percent of maximum LDH release from lysed cells minus baseline.

Halo-Mt colocalization with Translocase of Outer Mitochondrial Membrane 20 (TOM20): Primary human astrocytes were transduced to express Halo-Mt and then plated onto poly-d-lysine coated glass coverslips. Cells were fixed in 2% paraformaldehyde/PBS, permeabilized in 0.01% TritonX-100, and blocked in solution described in [72]. Cells were incubated overnight at 4°C with a 1:500 dilution of rabbit anti-HaloTag (Promega, #G9281) and 1:100 dilution of mouse anti-TOM20 (Santa Cruz Biotechnology, #sc-17764) in block solution, washed, then incubated in 1:750 dilution of Alexa Fluor 488-conjugated goat anti-rabbit (CST, #4412) and 1:200 dilution of Alexa Fluor 647-conjugated goat anti-mouse (Invitrogen, #A32728). Cells were then washed three times in PBS, and mounted onto glass slides with ProLong Diamond Antifade Mountant with DAPI. Individual cells were imaged on the EVOS M5000S (Thermo Fisher Scientific, Fair Lawn NJ) imaging system with the 60x objective 1.4na.

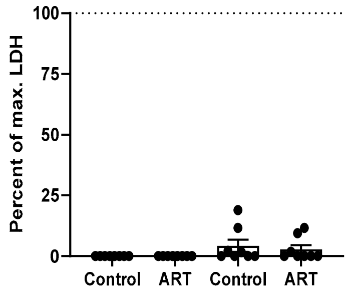

Figure S1. ART is not cytotoxic to astrocytes. Supernatants were collected from astrocytes treated for 24 h or daily for 7 days with ART, and LDH was measured. Graph shows the mean percent of LDH from supernatants from control and ART treated cells after each time point, relative to the maximum LDH released from a set of lysed cells included in each independent experiment. Error bars signify SEM. n=8.

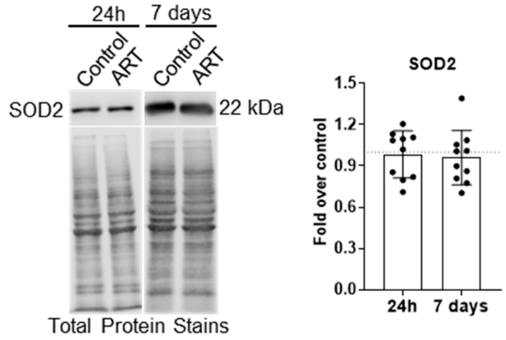

Figure S2. ART does not change SOD2 in astrocytes. Primary human astrocytes were treated for 24h or daily for 7 days with ART, and SOD2 western blotting was performed. Representative SOD2 western blots after 24h and 7 days of daily ART treatment are shown. Graph shows the mean fold changes of SOD2 relative to control represented by the dashed line at 1. Error bars represent SD. n=10.

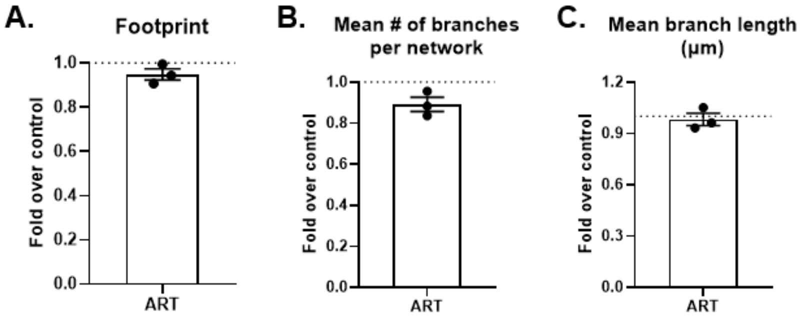

Figure S3. ART does not alter the mitochondrial network. Primary human astrocytes expressing Halo-Mt were treated daily for 7 days with ART, exposed to HaloTag ligand, fixed, and then immunolabeled with LAMP1. Cells were imaged by confocal microscopy for colocalization studies, then MiNA was performed as described in Methods on the same images. Graphs are the mean fold change of ART over control, shown by the dashed line at 1, for (A) the mean area of mitochondria normalized to cell area (Footprint, arbitrary units), (B) the mean number of branches per individual mitochondrial network, and (C) the mean branch length (µm) of all lines representing mitochondrial structures. Error bars represent SEM. n=3 independent experiments, with a total of 60-68 cells per condition analyzed.

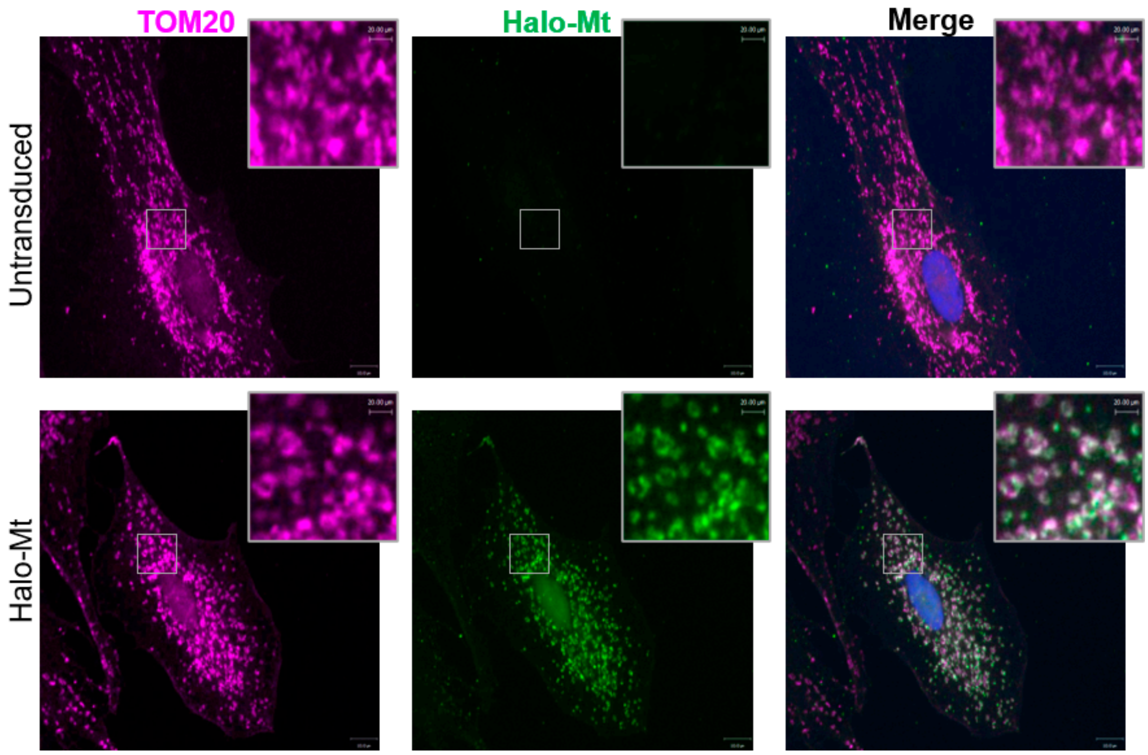

Figure S4. Halo-Mt localizes properly to the mitochondrial membrane. Astrocytes were transduced to express Halo-Mt, then immunolabeled for Halo-Mt and TOM20, an outer mitochondrial membrane protein. Representative images demonstrating colocalization of Halo-Mt (green) with TOM20 (magenta). Blue is nucleus. Scale bar =100 μM. Insets are enlarged views of the boxed areas, highlighting mitochondrial morphology (left panels), and colocalization (right panels). Inset scale bar=20 μM.

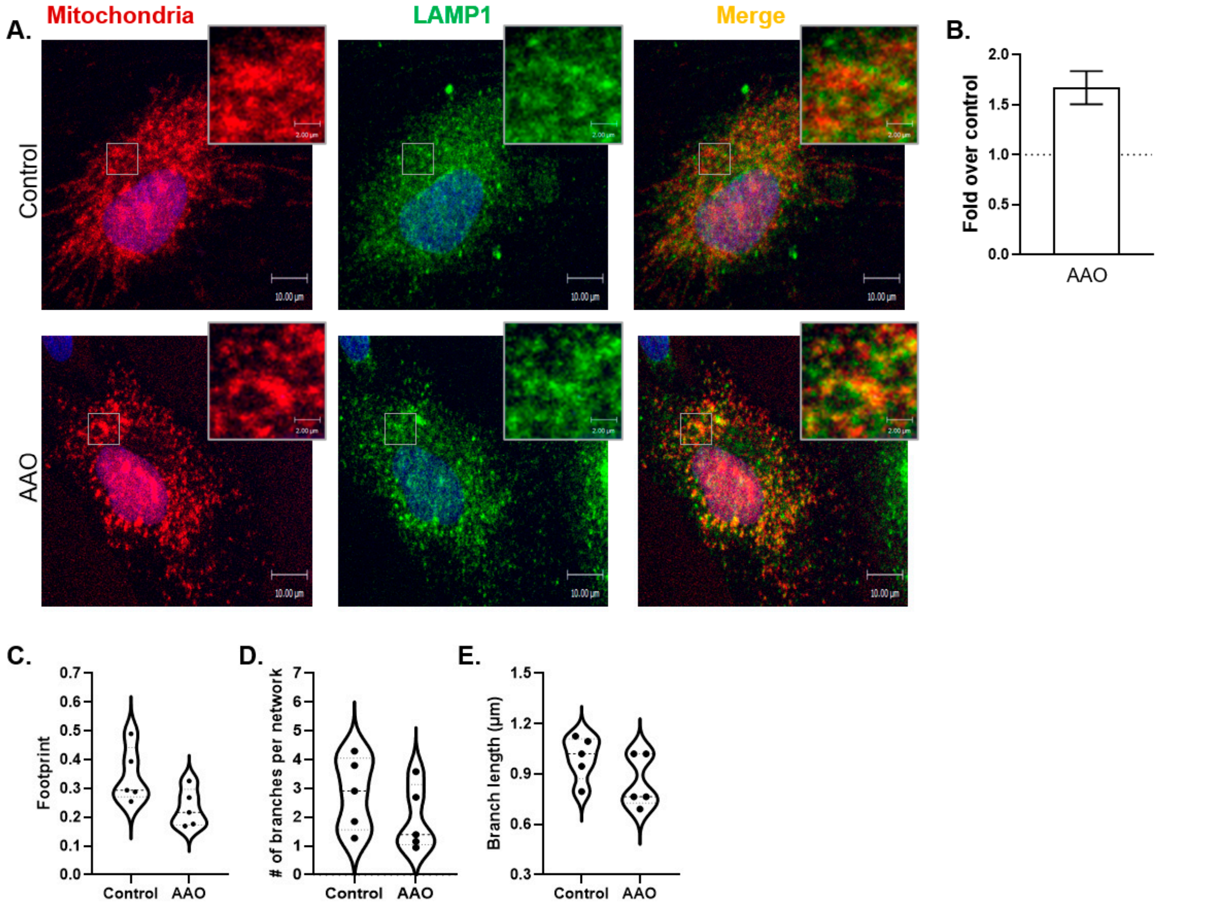

Figure S5. Mitophagic flux can be detected using Halo-Mt, and Antimycin A plus Oligomycin O reduce the size of the mitochondrial network. Halo-Mt transduced astrocytes were treated for 4 h with 10 µM Antimycin plus 10 µM Oligomycin A (AAO). Cells were then exposed to HaloTag ligand, fixed, and immunolabeled for LAMP1. Cells were imaged by confocal microscopy, and analyzed for (A-B) mitochondrial colocalization with LAMP1, and (C-E) Mitochondrial Network Analysis (MiNA) was performed as in Methods. (A) Representative images of control and AAO-treated cells. Red=mitochondria; green (pseudocolor)=lysosomes; blue=nucleus. Scale bar =10 µM. Insets are enlarged views of the boxed areas, highlighting mitochondrial morphology (left panels), and colocalization (right panels). Inset scale bar=2 µM. (B) Mean fold change of the volume of red colocalized with green, normalized to total green volume in AAO-treated cells relative to control cells shown by the dashed line at 1. Error bars represent SEM; n=1, with a total of 8-10 cells per condition analyzed. (C-E) Violin plots of MiNA show (C) the mean area of mitochondria normalized to cell area (Footprint, arbitrary units), (D) the mean number of branches per individual mitochondrial network, and (E) the mean branch length (µm) of all lines representing mitochondrial structures. n=1, with 5 cells analyzed per condition.
